# Association between menopausal hormone therapy, mammographic density and breast cancer risk: results from the E3N cohort study

**DOI:** 10.1186/s13058-021-01425-8

**Published:** 2021-04-17

**Authors:** M. Fornili, V. Perduca, A. Fournier, A. Jérolon, M. C. Boutron-Ruault, G. Maskarinec, G. Severi, L. Baglietto

**Affiliations:** 1 Department of Clinical and Experimental Medicine, University of Pisa, Pisa, Italy; 2 Laboratoire MAP 5 (UMR CNRS 8145), Université de Paris, Paris, France; 3 University Paris-Saclay, UVSQ, Inserm, Gustave Roussy, “Exposome and Heredity” team, CESP UMR1018, 94805 Villejuif, France; 4 University of Hawaii Cancer Center, Honolulu, USA; 5 Department of Statistics, Computer Science and Applications (DISIA), University of Florence, Florence, Italy

**Keywords:** Mammographic density, Menopausal hormone therapy, Menopause, Breast cancer risk, Mediation analysis

## Abstract

**Background:**

Menopausal hormone therapy (MHT) is a risk factor for breast cancer (BC). Evidence suggests that its effect on BC risk could be partly mediated by mammographic density. The aim of this study was to investigate the relationship between MHT, mammographic density and BC risk using data from a prospective study.

**Methods:**

We used data from a case-control study nested within the French cohort E3N including 453 cases and 453 matched controls. Measures of mammographic density, history of MHT use during follow-up and information on potential confounders were available for all women. The association between MHT and mammographic density was evaluated by linear regression models. We applied mediation modelling techniques to estimate, under the hypothesis of a causal model, the proportion of the effect of MHT on BC risk mediated by percent mammographic density (PMD) for BC overall and by hormone receptor status.

**Results:**

Among MHT users, 4.2% used exclusively oestrogen alone compared with 68.3% who used exclusively oestrogens plus progestogens. Mammographic density was higher in current users (for a 60-year-old woman, mean PMD 33%; 95% CI 31 to 35%) than in past (29%; 27 to 31%) and never users (24%; 22 to 26%). No statistically significant association was observed between duration of MHT and mammographic density. In past MHT users, mammographic density was negatively associated with time since last use; values similar to those of never users were observed in women who had stopped MHT at least 8 years earlier. The odds ratio of BC for current versus never MHT users, adjusted for age, year of birth, menopausal status at baseline and BMI, was 1.67 (95% CI, 1.04 to 2.68). The proportion of effect mediated by PMD was 34% for any BC and became 48% when the correlation between BMI and PMD was accounted for. These effects were limited to hormone receptor-positive BC.

**Conclusions:**

Our results suggest that, under a causal model, nearly half of the effect of MHT on hormone receptor-positive BC risk is mediated by mammographic density, which appears to be modified by MHT for up to 8 years after MHT termination.

**Supplementary Information:**

The online version contains supplementary material available at 10.1186/s13058-021-01425-8.

## Background

Mammographic density, that is the dense area (DA) of the breast consisting of epithelial and stromal tissue that appears light on a mammogram—as opposed to fat tissue that appears dark (non-dense area, NDA)—is one of the strongest risk factors for breast cancer (BC) in both pre- and postmenopausal women. A meta-analysis of 13 case-control studies estimated a 40% and 50% increased risk for one standard deviation increase of absolute DA and percent DA relative to the whole area of the breast, respectively [1]. Longitudinal studies have shown that mammographic density decreases with age, with the strongest decline occurring at the menopausal transition [2–4]. Menopausal hormone therapy (MHT) can be prescribed to women to balance oestrogen depletion occurring at menopause [5]. One of the most concerning side effects of MHT is the increased risk of hormone-related cancers, including breast (for oestrogen-progestogen MHT) and endometrial cancer (for oestrogen alone MHT) [6]. A recent pooled analysis on 58 studies has concluded that MHT increases BC risk of current users even during the first 1–4 years of therapy and that the increased risk still persists 10 years after stopping the therapy [7]. The same pooled analysis has showed an increased risk associated with either oestrogen plus progestogen and oestrogen alone, with a higher effect of the former formulation.

A recent systematic literature review has identified 22 studies reporting the association of MHT with mammographic density, including 16 observational studies and 6 randomized trials [8]: mammographic density was higher among ever compared to never MHT users, with the highest values observed among current users. Also, the association of MHT with mammographic density was stronger for oestrogen plus progestogen use than for oestrogens alone [9]. Evidence from published studies suggests that the proportion of the effect of MHT on BC risk mediated by its action on mammographic density varies from 10 to 22% [10, 11].

The aim of our work was to study the association between patterns of use of MHT (use, duration and time since last use) and mammographic density to better understand their independent and mediated effect on BC risk, overall and by hormone receptor status. For our analyses, we have used data on postmenopausal women from a BC case-control study nested within the French cohort E3N [12].

## Methods

### Study population

The French E3N cohort comprises 98,995 women insured by a national health insurance scheme covering mostly teachers (Mutuelle Générale de l’Education Nationale). Since recruitment in 1990, women that were aged between 40 and 65 years at baseline were followed-up every 2–3 years with self-administered, structured questionnaires aimed at collecting sociodemographic, reproductive and lifestyle characteristics of participants together with their health conditions [13]. Most breast cancer cases were self-reported in the questionnaires or, to a lesser extent, spontaneously reported by participants’ next-of-kin, or identified from cause of death data. Pathology reports were obtained for 95% of the incident cases identified in the entire cohort and were used to confirm the cases and to extract information on tumour characteristics such as stage, grade, hormonal receptor status and histological type.

Based on 5557 BC cases of invasive adenocarcinoma of the breast (International Classification of Disease for Oncology codes C50.0-C50.9) diagnosed between baseline and the end of 2008, a nested case-control study was designed to investigate the association between mammographic density and BC risk.

Details of the nested case-control study are provided elsewhere [12]. Briefly, 920 invasive adenocarcinomas of the breast diagnosed between 1990 and 2010 with known laterality and at least one mammogram taken between baseline and diagnosis were matched using a density sampling procedure to women of the cohort BC-free at the age at diagnosis of the corresponding case (reference age); matching factors also included year of birth (± 3 years) and menopausal status at baseline. Mammograms were retrieved for women in the nested case-control study; the closest mammogram prior to the reference age was identified and used to quantify mammographic density (index mammogram). Matched pairs were excluded if the difference between the age at mammogram of the case and the matched control was more than 5 years (97 case-control pairs) or if one of the women within the pair was missing information for BMI at the time of mammogram (3 pairs). From the remaining 820 case-control pairs, for the present study, we selected the 906 women (453 case-control pairs) older than 55 years at the date of the index mammogram; the age of 55 years was chosen as proxy for menopausal status.

### Assessment of mammographic density

For each matched case-control pair, mammographic density was quantified from the image of the breast where the tumour was diagnosed for the case and of the ipsilateral breast for the matched control. Given the high correlation between the mammographic density measures obtained from the craniocaudal and mediolateral projections [14] and consistent with the majority of the previous studies [1], the craniocaudal images of the breast were used. The mammographic films were digitized with an Array 2905 high-density film digitizer (Array Corporation Europe, Roden, The Netherlands) with a resolution of 300 PPI and were resized for density reading with a proportional maximal size of 800 × 400 pixels. A single reader (GM), who was blinded to case-control status, assessed total breast area and DA in batches of 200 mammograms using a computer-assisted technique (Cumulus, Sunnybrook Health Sciences Centre, University of Toronto, Toronto, Canada) [15]. Percent mammographic density (PMD) was computed as the ratio of DA to the total breast area, and NDA as the difference between total breast area and DA. For quality control, a random sample of 120 images was read in duplicate with resulting intraclass correlation of 0.98 for total breast area, 0.95 for DA, and 0.96 for PMD.

### Characteristics of the study sample

The pattern of use of MHT during the follow-up until the mammogram, including status of use at mammogram (*never* versus *current* versus *past MHT users*), formulation of MHT, duration of use for ever users and time since last use for past users, was calculated from data collected from the baseline and follow-up questionnaires. Formulation of MHT was coded according to the history of MHT use reported by the women through the repeated questionnaires as oestrogen alone (if use of oestrogen plus progestogen was never reported), oestrogen plus progestogens (if use of oestrogen alone was never reported) and other (any other MHT formulation).

Following previous findings in the E3N cohort [16], we further distinguished users of oestrogens plus progestogens in users of *oestrogens plus progesterone or dydrogesterone*, users of *oestrogens plus any other progestogens* and *users of both* formulations. Age at menarche was coded as *less than 12 years*, *12 years* and *more than 12 years*. Oral contraceptive use was defined as *ever* versus *never*; parity and lactation were combined in a single variable coded as *nulliparous* versus *parous with no lactation* versus *parous with lactation for less than 4 months* versus *parous with lactation for 4 months or more*; family history of breast cancer in first-degree relatives was coded as *yes* versus *no*; BMI at time of the mammogram was assigned according to the value reported in the questionnaire closest to the mammogram.

### Statistical methods

In order to achieve normality of the distribution of PMD, DA and NDA, the square root transformation was applied to the original variables. The effect of MHT on each mammographic density measure was estimated by fitting age-adjusted linear regression models to the transformed mammographic density variables. First, we fitted models where MHT was categorized as never, current and past users at the time of the mammogram; then, to account for pattern of use, duration for “ever users” and time since last use for “past users” were dichotomized according to their medians. Finally, we fitted polynomial models with duration and time since last use as continuous variables; the degree of the polynomial best fitting the data was identified through Akaike’s Information Criterion (AIC). For each linear model, the association of MHT with each mammographic density measure was assessed with the *F*-test. In the linear regression analyses, to account for the over-representation of BC cases in the data set compared to the general population, cases and controls were weighted by *p*/2 and (1 − *p*)/2 respectively [17], where the probability *p* was set to 0.08, an estimate of the prevalence of BC cases in the general population of women aged more than 55 years. The effect of the following potential confounders was evaluated: family history of BC in first-degree relatives, age at menarche, previous use of oral contraceptives, parity and lactation. Because BMI was considered as a mediator of the effect of MHT on mammographic density, it was not included among the potential confounders. The heterogeneity of effect of the type of MHT formulation was assessed by comparing ever users of a single type of hormonal therapy to never users, after excluding women who used more than one type of MHT.

To estimate the total effect of MHT on BC risk and the component of the effect mediated by PMD, we adopted two different approaches. First, the odds ratios (ORs) for BC were estimated from the unconditional logistic model adjusted for the matching variables (i.e. reference age, year of birth, menopausal status at baseline) and BMI (partially adjusted model); the mediated effect was calculated as the difference between the coefficients of MHT from the models without and with PMD [18]. The additional effect of family history of BC, age at menarche, use of oral contraceptive, parity and lactation as potential additional confounders was also evaluated (fully adjusted model). In order to exclude the possibility that the differences between the results of the partially and fully adjusted mediation models were due the presence of missing values, both analyses were performed after excluding 48 case-control pairs with missing values in any of the potential confounders. Second, to account for the correlation between PMD and BMI in evaluating their joint role as mediators of the effect of MHT on BC risk, we applied a modified version of the quasi-Bayesian algorithm by Imai et al. [19] described elsewhere [20]. In this model, the linear relationship between the squared root transformed PMD and MHT was estimated using the weights to account for the over-representations of BC cases, as described above. The mediation analyses were conducted for all types of BC combined and separately for hormone receptor-positive BC (ER positive or PR positive) and hormone receptor-negative BC (ER negative and PR negative). For all models, the proportion of effect mediated by PMD was calculated on the logarithmic scale (log (OR_mediated_)/log (OR_total_)) [13].

## Results

For the 453 cases and 453 controls of the study sample, the median time between the enrolment in the E3N cohort and mammogram was 11.3 years (interquartile range (IQR) 9.0 to 13.2 years). Fifty percent of the women were born before 1939 (IQR 1936 to 1943); median age at diagnosis of cases was 61 years (IQR 59 to 66); the median age at mammogram was 61 years (IQR 58 to 65 years); the median time between mammogram and diagnosis was 0.2 years (IQR 0.1 to 1.6 years). Cases had higher BMI and DA than controls (median BMI 23.1 versus 22.8 kg/m^2^, *P* = 0.005; median DA: 35 versus 29 cm^2^, *P* < 0.001); no statistically significant difference was observed for NDA (median NDA 68 versus 72 cm^2^, *P* = 0.36). The proportion of MHT ever users was higher among cases than controls (0.83 vs 0.76, *P =* 0.009). Among MHT users, 4.2% used exclusively oestrogen alone compared with 68.3% who used exclusively oestrogens plus progestogens (19.2% used either oestrogens plus progesterone or dydrogesterone, 33.3% used oestrogens plus progestogens other than progesterone or dydrogesterone, 15.8% used both). Other characteristics of the study sample are reported in Table 1.

**Table 1:** Characteristics of the women

Characteristic	All (***N*** = 906)	Controls (***N*** = 453)	Cases (***N*** = 453)
Reference age (years)*	61 (59 to 66)	61 (59 to 66)	61 (59 to 66)
Age at mammogram (years)*	61 (58 to 65)	61 (58 to 65)	60 (58 to 65)
*MHT at mammogram, N (%)*			
Never	189 (20.9)	110 (24.3)	79 (17.4)
Current	432 (47.7)	217 (47.9)	215 (47.5)
Past, 0–2 years since last use	154 (17.0)	50 (11.0)	104 (23.0)
Past, > 2 years since last use	131 (14.5)	76 (16.8)	55 (12.1)
*Type of MHT at mammogram, N (%)*			
None	189 (20.9)	110 (24.3)	79 (17.4)
Oestrogen	30 (3.3)	15 (3.3)	15 (3.3)
Oestrogens plus progestogens	490 (54.1)	233 (51.4)	257 (56.7)
*Oestrogen plus progesterone or dydrogesterone*	138 (15.2)	79 (17.4)	59 (13.0)
*Oestrogen plus progestins*	239 (26.4)	99 (21.9)	140 (30.9)
*Both*	113 (12.5)	55 (12.1)	58 (12.8)
Others	197 (21.7)	95 (21.0)	102 (22.5)
*BMI at mammogram (kg/m*^*2*^)*	22.9 (21.1 to 25.1)	22.8 (21.0 to 24.8)	23.1 (21.3 to 25.4)
PMD*	32 (20 to 45)	31 (17 to 43)	35 (23 to 49)
*DA (cm*^*2*^)*	33 (20 to 47)	29 (17 to 42)	35 (23 to 52)
*NDA (cm*^*2*^)*	70 (47 to 97)	72 (49 to 97)	68 (46 to 96)
ER and PR status, N (%)**			
ER+ and PR+			224 (60.6)
ER+ and PR−			77 (20.8)
ER− and PR+			13 (3.5)
ER− and PR−			56 (15.1)
*Family history of breast cancer in first-degree relatives, N (%)*			
No	771 (85.1)	394 (87.0)	377 (83.2)
Yes	135 (14.9)	59 (13.0)	76 (16.8)
*Age of menarche (years), N (%)*			
< 12	162 (17.9)	69 (15.2)	93 (20.5)
12	244 (26.9)	125 (27.6)	119 (26.3)
> 12	500 (55.2)	259 (57.2)	241 (53.2)
*Past use of oral contraceptives, N (%)*			
No	452 (49.9)	232 (51.2)	220 (48.6)
Yes	454 (50.1)	221 (48.8)	233 (51.4)
Parity and lactation, N (%)**			
Nulliparous	116 (13.6)	59 (14.0)	57 (13.3)
Parous without lactation	205 (24.1)	94 (22.3)	111 (25.8)
Parous with lactation for less than 4 months	246 (28.9)	123 (29.2)	123 (28.6)
Parous with lactation for 4 months or more	284 (33.4)	145 (34.4)	139 (32.3)

For each matched case-control pair, reference age is the age at diagnosis of the case

*MHT* menopausal hormone therapy, *BMI* body mass index, *PMD* percent mammographic density, *DA* dense area, *NDA* non-dense area, *ER* oestrogen receptor, *PR* progesterone receptor

*Median (interquartile range)

**Number of missing: ER/PR status, 83; Parity and lactation, 55

Compared to the whole E3N cohort, the control group of this study sample had higher proportions of women who at baseline never used MHT (87.4% versus 78.8%), never used oral contraceptives (51.2% versus 45.3%), had a BMI lower than 25 kg/m^2^ (88.1 versus 82.2), breastfed for more than 4 months (40% versus 22%) and had a family history of breast cancer in first-degree relatives (13.0% versus 8.1%) (Supplementary Table S1).

### Association between menopausal hormone therapy and mammographic density

MHT use status at mammogram was significantly associated with PMD, DA and NDA (*P* < 0.001, < 0.001 and 0.006, respectively): for PMD and DA, past users had significantly higher values than never users (*P =* 0.003 and *P =* 0.005 respectively) and significantly lower values than current users (*P =* 0.004 and *P =* 0.003 respectively); for NDA, both past and current users had lower levels than never users but differences were statistically significant only for current versus never users (*P =* 0.001). Table 2 shows the predicted mammographic density measures by MHT status for women aged 60 and 70 years. Distinguishing MHT ever users by type of MHT formulation (*oestrogen* versus *oestrogen plus progesterone or dydrogesterone* versus *oestrogen plus other progestogens*) did not improve the fit for any of the mammographic density variables (*P =* 0.92, 0.86 and 0.67, for PMD, DA and NDA respectively).

**Table 2:** Predicted mammographic measures in women 60 and 70 years old by menopausal hormone therapy use

	PMD (%)	DA (cm^2^)	NDA (cm^2^)
	Predicted (95% CI)	Predicted (95% CI)	Predicted (95% CI)
*For a woman aged 60 years*			
Never	24 (22 to 26)	24 (22 to 26)	78 (72 to 83)
Past use	29 (27 to 31)	29 (26 to 31)	71 (66 to 76)
Current use	33 (31 to 35)	33 (31 to 35)	67 (63 to 70)
*For a woman aged 70 years*			
Never	20 (18 to 23)	24 (21 to 27)	94 (87 to 101)
Past use	25 (22 to 27)	29 (26 to 32)	87 (80 to 93)
Current use	29 (26 to 32)	33 (30 to 37)	82 (75 to 89)

*PMD* percent mammographic density, *DA* dense area, *NDA* non-dense area, *MHT* menopausal hormone therapy

To assess the effect of pattern of use of MHT on mammographic density, we fitted a model that included both duration and time since last use categorized according to the medians in all women (6 and 2 years, respectively). Among past users, time since last use was negatively associated to PMD (*P =* 0.009) and DA (*P* < 0.001). There was no statistically significant association of duration with PMD, DA, nor NDA (all *P* > 0.05). For none of the mammographic density variables, adding the interaction between duration and time since last use significantly improved the model. Duration was therefore excluded from the model. Table 3 reports the corresponding predictions of PMD, DA and NDA for women aged 60 and 70 years. For all three mammographic density variables, the values for past users who stopped MHT less than 2 years earlier were not statistically significantly different from those of current users (*P =* 0.65 for PMD, 0.88 for DA and 0.95 for NDA), whereas the values for past users who stopped more than 2 years earlier were not significantly different from those of never users (*P =* 0.19, 0.39 and 0.44, respectively).

**Table 3:** Predicted mammographic measures in women 60 and 70 years old by pattern of menopausal hormone therapy use

	PMD (%)	DA (cm^**2**^)	NDA (cm^**2**^)
	Predicted (95% CI)	Predicted (95% CI)	Predicted (95% CI)
*For women aged 60 years*			
Never	24 (22 to 26)	24 (22 to 26)	78 (72 to 83)
Current	33 (31 to 35)	33 (31 to 35)	67 (63 to 70)
Past, 0–2 years since last use	32 (29 to 36)	33 (29 to 37)	67 (60 to 74)
Past, > 2 years since last use	26 (24 to 29)	25 (23 to 28)	74 (68 to 81)
*For women aged 70 years*			
Never	20 (18 to 23)	24 (21 to 27)	94 (86 to 101)
Current	29 (26 to 32)	33 (30 to 37)	82 (75 to 89)
Past, 0–2 years since last use	28 (24 to 32)	33 (29 to 38)	81 (73 to 91)
Past, > 2 years since last use	23 (20 to 26)	26 (23 to 29)	90 (82 to 98)

*PMD* percent mammographic density, *DA* dense area, *NDA* non-dense area

**Fig. 1:**
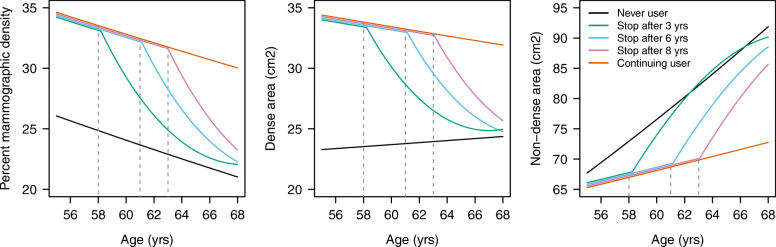
Trends of mammographic measures for different patterns of use of menopause hormone therapy (MHT). Trends by age of percent mammographic density, dense area and non-dense area for a hypothetical woman who never used MHT (black); one who started MHT use at the age of 55 and never stopped (orange); one who started at the age of 55 and stopped at the age of 58 (green); one who started at the age of 55 and stopped at the age of 61 (blue); and one who started at the age of 55 and stopped at the age of 63 (magenta). Lines are from models including age at mammogram, MHT status (never vs ever), duration of use for ever users (continuous linear) and time since last use for past users (continuous quadratic)

For all three mammographic density variables, the best polynomial model included the first-degree polynomial for duration and the second-degree polynomial for time since last use (supplementary Table S2). The trends by age of PMD, DA and NDA predicted by such models are shown in Fig. 1 for a woman who never used MHT; a woman who started MHT at the age of 55 and who never stopped; and a woman who started at the age of 55 and stopped after 3, 6, or 8 years (second, third and fourth quartiles of the MHT duration). It takes less than 1 year of use of MHT for mammographic density to reach a plateau, as indicated by the discontinuity of the predicted curve between never and current users. According to the models, for a woman who started MHT at 55 years and who stopped after 3 years, the levels of PMD, DA and NDA returned to the levels of never users after approximately 8, 9 and 4 years respectively; after 8, 11 and 6 years if the same woman stopped MHT after 6 years; and after 8, 12 and 6 years if she stopped after 8 years.

The adjustment for additional potential confounders did not materially change any of the above estimates.

### Mediation analysis

Considering that the effect of MHT on PMD can be observed for up to 8 years after MHT discontinuation (as reported in the previous section), we conducted mediation analyses only on current and never MHT users. In the model adjusted for age and for the matching variables, the OR of BC associated with *current* versus *never* use of MHT was 1.67 (95% CI, 1.04 to 2.68); when PMD was added into the model, the OR became 1.40 (0.86 to 2.28) that corresponds to a 34% mediated effect on the log scale. When mediation analysis was conducted stratifying by hormone receptor status, it appeared that the association between MHT and BC risk was mainly due to hormone receptor-positive breast cancers: the OR of hormone receptor-positive BC associated with current use of MHT was 1.81 (1.05 to 3.10); when PMD was added into the model, the OR became 1.46 (0.84 to 2.57), which corresponds to a 36% mediated effect on the log scale. For hormone receptor-negative BC, the association between MHT and BC was not significant, either without or with inclusion of PMD into the model (OR = 0.64, 0.15 to 2.81, and OR = 0.57, 0.12 to 2.70, respectively). Similar findings were obtained from the regression models adjusted for all potential confounders, where the proportion of the effect of MHT mediated by PMD was 40% for any BC and 41% for hormone receptor-positive BC.

Table 4 reports the results from the mediation model that accounted for the joint mediation effect of PMD and BMI (Pearson’s correlation coefficient between square-rooted transformed PMD and BMI − 0.39, *P* < 0.001). In the model adjusted for age and matching variables, the OR associated with current use of MHT was 1.46 (0.42 to 2.41); the average direct effect was 1.37 (0.85 to 2.18); the average mediated effects were 1.20 (1.06 to 1.41) through PMD and 0.90 (0.78 to 0.98) through BMI. When mediation analysis was stratified by hormone receptor status, it appeared that the association between MHT and BC risk was limited to hormone receptor-positive breast cancers: the total effect of MHT was 1.58 (0.93 to 2.64), resulting from a direct effect of 1.44 (0.85 to 2.34) and indirect effects of 1.22 (1.06 to 1.52) through PMD and 0.91 (0.78 to 0.99) through BMI. According to this model, the proportion of the effect of MHT mediated by PMD on the log scale was 48% for any BC and 43% for hormone receptor-positive BC.

**Table 4:** Mediation analysis of the effect of current versus never use (reference category) of menopausal hormone therapy on breast cancer risk, overall and by ER and PR status. The table reports the OR and 95% confidence intervals from the unconditional logistic models adjusted for age at mammogram and the matching variables (reference age, year of birth and menopausal status at baseline)

	All OR (95% CI)	ER+ or PR+ OR (95% CI)	ER− and PR− OR (95% CI)
	*N*_*CA*_ = 196/*N*_*CO*_ = 196	*N*_*CA*_ = 150/*N*_*CO*_ = 150	*N*_*CA*_ = 22/*N*_*CO*_ = 22
Total effect	1.46 (0.92 to 2.41)	1.58 (0.93 to 2.64)	0.63 (0.11 to 2.61)
Average direct effect	1.37 (0.85 to 2.18)	1.44 (0.85 to 2.34)	0.61 (0.10 to 2.48)
Average joint mediated effect	1.07 (0.92 to 1.25)	1.10 (0.93 to 1.37)	1.00 (0.48 to 2.52)
Average mediated effect by PMD	1.20 (1.06 to 1.41)	1.22 (1.06 to 1.52)	1.10 (0.61 to 2.63)
Average mediated effect by BMI	0.90 (0.78 to 0.98)	0.91 (0.78 to 0.99)	1.00 (0.49 to 1.40)

Mediation analysis evaluating the direct and indirect effect of current versus never use of MHT on breast cancer risk, overall and by ER and PR status in presence of PMD and BMI, correlated mediators

No material changes were observed in the results of the mediation analysis when the models were adjusted for all potential confounders (results not shown). Qualitatively similar results were observed for MHT coded as ever versus never (supplementary Table S3).

## Discussion

Our analysis of the data collected within the E3N prospective cohort showed that postmenopausal women taking MHT had a higher PMD and DA and a lower NDA than never users of MHT; this difference was observed already in women who used MHT for at least 1 year. In past users, mammographic density levels were lower than in current users and a negative association was observed with time since last use; values similar to those of never users were observed in women having stopped MHT for at least 8 years. The effect of current use of MHT on BC risk was partially direct and partially mediated by PMD; the mediated effect was restricted to the hormone receptor-positive tumours. Given that among the MHT users only a minority used oestrogen alone, our findings were driven by the effect of oestrogens plus progestogen use. There was no evidence of heterogeneity by type of progestogens.

The association between MHT use and mammographic density has been previously reported [9, 21–23]. A nested case-control study within the Women’s Health Initiative randomized study estimated that after 1 year since starting therapy PMD increased of about ten percentage point in women in the oestrogen-progestin arm, whereas no change was observed in women taking placebo [21]; this longitudinal change was comparable to our estimate of a difference of 8 percentage points in PMD (estimated from the polynomial model) between a woman aged 56 years who took MHT for 1 year and a never user aged 55 years old. Our observation that women taking MHT for less than 1 year had higher mammographic density levels than never users and that the difference was observed also in those who stopped treatment less than 8 years earlier is consistent with the findings about the association between MHT and BC risk. Previous analyses of the full E3N cohort reported an effect of MHT on BC risk already in the first 2 years after starting the therapy [16, 24]. A meta-analysis on 58 studies estimated that the increased risk of BC associated with MHT in current users appeared in the first 5 years of use and almost doubled in the following 5–14 years of use; in past users, excess risk persisted even after 10 years since stopping the therapy [7].

According to our data, MHT modifies mammographic density similarly as it modifies BC risk, an observation consistent with the role of mammographic density as mediator of the effect of MHT on BC risk. The proportion of the effect of MHT on BC explained by mammographic density has been previously reported [10, 11]: the estimates range from the 11% obtained in the participants of the Danish diet, cancer and health cohort undergoing mammographic screening [11] to the 22% obtained in a case-control study nested within the NHS cohorts [10]. The latter estimate is similar to the 34% mediated effect that we obtained using the difference of coefficients. When we accounted for the correlation between mammographic density and BMI, we obtained a higher estimate of the proportion of the mediated effect of MHT on BC through mammographic density than applying the difference of coefficients approach (48 vs 34%). Our results contribute substantial evidence about the role of mammographic density as mediator of the effect of MHT on BC risk based so far on relatively sparse data.

In the meta-analysis by the Collaborative Group on Hormonal Factors in Breast Cancer, the increased risk of BC associated with MHT was observed for all type of MHT except vaginal oestrogen and the risk was greater for oestrogen plus progestogen than for oestrogen alone; also, the excess BC risk was higher for ER-positive than ER-negative BCs [7]. Previous analyses conducted on the entire E3N cohort found that the effect of MHT on BC risk depends on the type of MHT preparation and provided evidence for a differential effect on risk by BC subtype [16]. Our analysis did not find evidence for heterogeneity of the effect of MHT on mammographic density by formulation, although the number of women taking oestrogens alone was too small to achieve adequate statistical power.

In our study, the proportion of women who used oestrogen alone was much lower compared with the other European study where MHT was assessed at baseline (3% versus 12% respectively) [11]; the reason of this difference may be due to the fact that in the category “oestrogen alone” we included women who took exclusively oestrogen alone (i.e. never took oestrogens plus progestogen), distinguishing them from those who took exclusively oestrogen plus progestogen and from those who took both. As to the subtype of BC, we observed that PMD mediated the effect of MHT on BC risk only for hormone receptor-positive BCs, but not for hormone receptor-negative BCs. In our study sample, we did not observe any association between MHT and hormone receptor-negative BC, somehow consistently with the observation from the entire E3N cohort that MHT had a stronger effect on ER-positive than ER-negative BC [16]. However, the small number of hormone receptor-negative BCs does not allow to formulate any reliable conclusion about the mediation effect of BC on hormone receptor-negative BC risk.

The main strengths of our study were its prospective design and the completeness of information about MHT pattern of use over a median period of time of 11 years. The richness of the information available from the E3N cohort allowed us to adjust for all known potential confounders of the relations between MHT, mammographic density and BC risk, essential for an unbiased estimate of the mediated causal effect [25]. Limitations of our study include the relatively small sample size and the self-reported information about the potential breast cancer risk factors. Good agreement has been found between self-reported and external measures of menopausal status and anthropometric measure in the E3N cohort, but misreporting of some reproductive history variables, such as age at menarche and lactation, might have occurred. The issues related to the representativeness of the E3N cohort of the general population are common to all prospective cohorts of volunteers but in general this should not affect the results of association studies [26]. Compared to the whole E3N cohort, the controls in our study sample had a slightly different distribution of the reproductive and anthropometric breast cancer risk factors, resulting in a lower breast cancer risk profile. Also, controls in our study had a higher proportion of women with a positive history of breast cancer in first-degree relatives compared with the whole cohort. These differences may be due to the fact that controls were sampled from participants with mammograms available, a group that is likely to include a higher proportion of women with a healthy lifestyle and with a more health-conscious attitude than the entire cohort, as previously reported [27]. Another limitation of our study is the small number of women taking oestrogen alone MHT that limited statistical power for comparisons between oestrogen alone and oestrogen plus progestogen MHT. Finally, repeated longitudinal measures of mammographic density were not available, and estimates of intra-individual changes in mammographic density over time were therefore not possible. Therefore, we could not quantify the proportion of effect of MHT on breast cancer risk mediated by change in mammographic density induced by MHT. In the ancillary study on mammographic density conducted within the Women Health Initiative, it was estimated that the change in percent mammographic density occurring within 1 year of treatment with oestrogen plus progestin therapy mediated 100% of the effect of the therapy on BC risk [10].

The use of MHT in Western countries strongly increased in the 1990s when studies suggested its beneficial effect on postmenopausal women’s health [28–31]. After the first results from the Women’s Health Initiative study in 2002 [32] reporting an excess risk of BC and cardiovascular diseases in the oestrogen plus progestin arm compared to the placebo arm, the number of MHT consumers abruptly decreased. A subsequent reanalysis of the follow-up data of the same trial and independent studies suggested that the benefits of MHT taken over menopause (e.g. improved overall survival) overcome its negative effects [33–36], but a general consensus on this has not been reached yet [37]. It has been estimated that in 2010 there were about 12 million users in Western countries [7]. The meta-analysis by the Collaborative Group on Hormonal Factors in Breast Cancer published in 2019 provided new elements for the ongoing debate about the safety of use of MHT [7, 38].

## Conclusions

Mammographic density levels were higher in current than never MHT users already within the first year of use, whereas levels in women who stopped therapy more than 8 years earlier were similar to levels of never users. Mammographic density mediated up to 50% of the effect of MHT on breast cancer risk.

Our results, if confirmed by independent longitudinal studies, indicate that MHT should be prescribed with caution particularly in women with high mammographic density and suggest that monitoring mammographic density during MHT use might be a useful strategy in situations when MHT prescription is appropriate.

## Supplementary Information

**Additional file 1:.** Supplementary Table S1. Characteristics of the study sample in comparison with the whole E3N cohort. Supplementary Table S2. Values of the Akaike Information Criterion (AIC) for regression models with the square root of percent mammographic density (PMD), dense area (DA) and non-dense area (NDA) as polynomial functions of duration of MHT use and time since last use. Supplementary Table S3. Mediation analysis of the effect of ever versus never use (reference category) of menopausal hormone therapy on breast cancer risk, overall and by ER and PR status. The table reports the OR and 95% confidence intervals from the unconditional logistic models adjusted for age at mammogram and the matching variables (reference age, year of birth and menopausal status at baseline).

## Data Availability

The dataset analysed during the current study are available from the corresponding author on reasonable request.

## Abbreviations

AIC: Akaike’s Information Criterion
BC: Breast cancer
DA: Dense area
CI: Confidence interval
IQR: Interquartile range
MHT: Menopausal hormone therapy
ND: Non-dense area
OR: Odds ratio
PMD: Percent mammographic density
