# Supplementary material for: Association between menopausal hormone therapy, mammographic density and breast cancer risk: results from the E3N cohort study
Source: Breast Cancer Res. 2021 Apr 17;23:47. doi: 10.1186/s13058-021-01425-8 (PMC8053286; doi:10.1186/s13058-021-01425-8)
Supplement: Supplementary file 1 — Additional file 1:. Supplementary Table S1. Characteristics of the study sample in comparison with the whole E3N cohort. Supplementary Table S2. Values of the Akaike Information Criterion (AIC) for regression models with the square root of percent mammographic density (PMD), dense area (DA) and non-dense area (NDA) as polynomial functions of duration of MHT use and time since last use. Supplementary Table S3. Mediation analysis of the effect of ever versus never use (reference category) of menopausal hormone therapy on breast cancer risk, overall and by ER and PR status. The table reports the OR and 95% confidence intervals from the unconditional logistic models adjusted for age at mammogram and the matching variables (reference age, year of birth and menopausal status at baseline). [file 13058_2021_1425_MOESM1_ESM.zip › Supplementary Table S1.docx]

**Supplementary Table S1** Characteristics of the study sample in comparison with the whole E3N cohort.

| **Characteristic** | **Controls**  ***N*=453**  **(%)** | **Expected** | **Cases**  ***N*=453**  **(%)** | **E3N**  ***N*=98,995**  **(%)** | ***p-value*** |
| --- | --- | --- | --- | --- | --- |
| *Cohort of birth* |  |  |  |  | *<0.001* |
| 1925-1929 | 11 (2.4) | 36 | 11 (2.4) | 7,808 (7.9) |  |
| 1930-1934 | 77 (17.0) | 59 | 78 (17.2) | 12,786 (12.9) |  |
| 1935-1939 | 145 (32.0) | 86 | 147 (32.5) | 18,742 (18.9) |  |
| 1940-1944 | 162 (35.8) | 109 | 160 (35.3) | 23,907 (24.2) |  |
| 1945-1949 | 58 (12.8) | 164 | 57 (12.6) | 35,752 (36.1) |  |
|  |  |  |  |  |  |
| *MHT at baseline, N (%)* |  |  |  |  | *<0.001* |
| Never | 396 (87.4) | 357 | 380 (83.9) | 67,899 (78.8) |  |
| Ever | 57 (12.6) | 96 | 73 (16.1) | 18,265 (21.2) |  |
|  |  |  |  |  |  |
| *BMI at baseline (kg/m^2^)* |  |  |  |  | *<0.001* |
| <=25 | 399 (88.1) | 372 | 381 (84.1) | 79,488 (82.2) |  |
| 25-30 | 51 (11.2) | 66 | 60 (13.2) | 14,123 (14.6) |  |
| >30 | 3 (0.7) | 14 | 12 (2.7) | 3,070 (3.2) |  |
|  |  |  |  |  |  |
| *Familiarity at baseline* |  |  |  |  | *0.003* |
| No | 394 (87.0) | 416 | 377 (83.2) | 86,013 (91.8) |  |
| Yes | 59 (13.0) | 37 | 76 (16.8) | 7,626 (8.1) |  |
|  |  |  |  |  |  |
| *Age of menarche (yrs)* |  |  |  |  | *0.57* |
| <12 | 69 (15.2) | 73 | 93 (20.5) | 15,619 (16.2) |  |
| 12 | 125 (27.6) | 136 | 119 (26.3) | 28,929 (30.0) |  |
| >12 | 259 (57.2) | 244 | 241 (53.2) | 51,975 (53.8) |  |
|  |  |  |  |  |  |
| *Use of oral contraceptives* |  |  |  |  |  |
| No | 232 (51.2) | 205 | 220 (48.6) | 44,811 (45.3) | *0.04* |
| Yes | 221 (48.8) | 248 | 233 (51.4) | 54,185 (54.7) |  |
|  |  |  |  |  |  |
| *Parity* |  |  |  |  |  |
| Nulliparous | 59 (13.0) | 63 |  | 12,176 (14.0) | *0.86* |
| Parous | 394 (87.0) | 390 |  | 74,441 (86.0) |  |
|  |  |  |  |  |  |
| *Lactation among parous* |  |  |  |  |  |
| Never | 94 (26.0) | 116 |  | 23,857 (32.0) | *<0.001* |
| Less than 4 months | 123 (34.0) | 166 |  | 34,257 (46.0) |  |
| 4 months or more | 145 (40.0) | 80 |  | 16,327 (22.0) |  |
